# Shifting Trends in Prostate Treatment: A Systematic Review Comparing Transurethral Resection of the Prostate and Holmium Laser Enucleation of the Prostate

**DOI:** 10.7759/cureus.46173

**Published:** 2023-09-29

**Authors:** Javed Iqbal, Yusra Mashkoor, Abdullah Nadeem, Sunanda Tah, Mouhammad Sharifa, Saroosh Ghani, Thanmai Reddy Thugu, Harshkumar Patel, Felicia T Bonner-Reid, Jeena Shrestha, Buure A Hassen

**Affiliations:** 1 Department of Neurosurgery, Mayo Hospital, Lahore, PAK; 2 Department of Internal Medicine, Dow University of Health Sciences, Karachi, PAK; 3 Department of Internal Medicine, Saint James School of Medicine, Arnos Vale, VCT; 4 Department of Internal Medicine, University of Aleppo, Aleppo, SYR; 5 Department of Medicine and Allied Sciences, Isra University, Hyderabad, PAK; 6 Department of Internal Medicine, Sri Padmavathi Medical College for Women, Tirupati, IND; 7 Department of Internal Medicine, Pandit Deendayal Upadhyay Medical College, Rajkot, IND; 8 Department of Internal Medicine, Universidad de Ciencias Médicas de Granma, Manzanillo, CUB; 9 Department of Internal Medicine, Jalalabad Ragib-Rabeya Medical College, Sylhet, BGD; 10 Department of Internal Medicine, Hayat Medical College, Addis Ababa, ETH

**Keywords:** quality care, prostatectomies, prostate, turp, holep

## Abstract

Our systematic review aimed to assess the effectiveness and suitability of holmium laser enucleation of the prostate (HoLEP) as a treatment for benign prostatic hyperplasia (BPH) in comparison to transurethral resection of the prostate (TURP). We analyzed 12 studies involving male participants aged 45-85 years, all of whom had BPH.

In our analysis, we compared HoLEP and TURP, with a focus on several primary outcomes, including postoperative International Prostate Symptom Score (IPSS), postvoid residual (PVR) volume, maximum flow rate (Qmax), and changes in sexual function post-treatment. HoLEP demonstrated advantages in certain aspects when compared to TURP.

HoLEP generally resulted in an improved postoperative IPSS in some studies, but not all studies showed a significant difference when compared to TURP. HoLEP was associated with improved Qmax in most studies, but one study found no significant difference between HoLEP and TURP. Patients who underwent HoLEP showed improvement in the PVR volume in some studies, while others found no significant change in the PVR volume with either HoLEP or TURP. Some studies reported a reduction in orgasm and ejaculatory scores following TURP, while no significant changes were observed in erectile function, intercourse satisfaction, and overall satisfaction scores. It is worth noting that previous reviews and meta-analyses had limited data on the effects of HoLEP and TURP on sexual dysfunction. TURP is associated with a higher risk of morbidity and mortality, which has led to its replacement with HoLEP as the gold standard for treating BPH, particularly due to its size-independent applicability. HoLEP also demonstrated greater efficacy in the postoperative period.

## Introduction and background

Benign prostatic hyperplasia (BPH) is a histological diagnosis characterized by an increase in the total number of prostatic glandular epithelial and stromal cells within the transition zone of the prostate [1-3]. Clinical BPH, on the other hand, is defined as an enlarged prostate, causing bladder outlet obstruction (BOO) that may eventually damage the bladder and the kidneys due to urine reflux, potentially resulting in obstructive nephropathy [4]. The prostate's transition zone surrounds the proximal urethra and has the potential for continued growth throughout life [5]. Consequently, BPH is closely linked to lower urinary tract symptoms (LUTS) due to the proximity of the affected zone to the urethra. These symptoms, such as dysuria, nocturia, frequency, urgency, difficulty initiating micturition, difficulty emptying the bladder, and a weak stream during micturition, are collectively referred to as LUTS [6]. BPH can cause LUTS through two mechanisms: (a) direct obstruction of urethral outflow (static component) and (b) enhanced smooth muscle tone within the enlarged gland (dynamic component) [7].

BPH is a common issue among the elderly population, significantly affecting their quality of life. The prevalence of BPH increases with age, with medical records suggesting that approximately 70 percent of men in the United States between the ages of 60 and 69 years, and 80 percent of men over 70 years old, experience some degree of BPH [8]. Prevalence rates rise from 8 percent in men aged 31 to 40 to 40 to 50 percent in men aged 51 to 60 and exceed 80 percent in men above the age of 80 [9]. Therefore, effective management of BPH is essential to alleviate patient discomfort and improve LUTS.

Numerous treatment options are available for BPH, encompassing lifestyle modifications, medical therapy, and surgical procedures. Lifestyle modifications include weight reduction, physical activity, limiting fluid intake before bedtime or traveling, and reducing the consumption of bladder irritants and diuretics, such as highly seasoned or irritative foods and caffeine or alcohol, respectively [10]. After lifestyle modifications, medical therapy serves as the first-line treatment, primarily involving alpha-blockers and 5 alpha-reductase inhibitors [11,12]. Invasive treatments are considered when prior therapies have proven ineffective and patients continue to experience symptoms. These invasive options include resection, ablation, or compression of prostatic tissue using various energy sources. Most procedures are performed via the urethra using a specialized cystoscope, with the exception of simple prostatectomy and prostatic artery embolization. The choice of treatment depends on factors like prostate gland size and shape, risk of bleeding, risk of sexual dysfunction, and patient preferences [13].

Transurethral resection of the prostate (TURP) remains the gold standard and an effective intervention for BPH [14]. The conventional monopolar TURP (M-TURP) has been utilized and studied for decades, with associated side effects [15]. These side effects prompted the introduction of a newer iteration of TURP known as bipolar TURP (B-TURP). B-TURP exhibits similar efficacy to M-TURP [16] but carries a significantly lower risk of side effects, including TUR syndrome [17]. Transurethral vaporization of the prostate (TUVP) represents an electrosurgical modification of the standard TURP procedure and may yield better results, including improved hemostasis, enhanced tissue visualization, and faster ablation [18-21]. Transurethral incision of the prostate (TUIP) involves incising the prostate at the bladder neck level to relieve LUTS [22]. Photoselective vaporization of the prostate or laser TURP (PVP) uses laser energy to vaporize prostatic tissue and is considered a safe option, particularly for elderly patients on chronic anticoagulation therapy [23-25]. Transurethral microwave therapy (TUMT) employs a specialized urethral catheter with an antenna to generate electromagnetic (EM) waves that induce localized heat changes. However, surgical retreatment rates may be higher compared to TURP [13]. Water vapor thermal therapy (WVTT) utilizes convective water vapor energy to ablate hypertrophic tissue [12,26]. Prostatic urethral lift (PUL) involves a nonablative approach to treat BPH through transprostatic tissue compression (Urolift System) [27,28]. Simple prostatectomy entails enucleating the prostatic tissue along with its capsule and is typically reserved for individuals with significantly enlarged prostates [7]. Other emerging techniques include transurethral needle ablation, robotic water jet treatment, prostate artery embolization, and laparoscopic and robotic prostatectomy [7,24].

Laser enucleation of the prostate utilizes energy from the holmium:yttrium-aluminum-garnet (YAG) laser or thulium: YAG laser to ablate prostatic tissue with minimal involvement of surrounding and deep tissues. These procedures are known as holmium laser enucleation of the prostate (HoLEP) and thulium laser enucleation of the prostate (ThuLEP). HoLEP can be employed in patients with BPH, irrespective of the prostate size [13]. It is associated with a shorter hospital stay, a shorter duration of catheter indwelling, and fewer bleeding complications while offering similar improvements in symptoms and urine flow rates compared to TURP [29]. HoLEP is also a viable treatment option for individuals on anticoagulation or antiplatelet therapy [30] and carries a lower risk of blood transfusion compared to TURP [31]. However, it does require a longer intraoperative time and is associated with a higher incidence of stress urinary incontinence [32,33]. In this systematic review, we will discuss the efficacy and outcomes of HoLEP in the context of BPH treatment, as well as the shift in the treatment trend toward HoLEP in comparison to conventional TURP.

## Review

Methods

**Figure 1:**
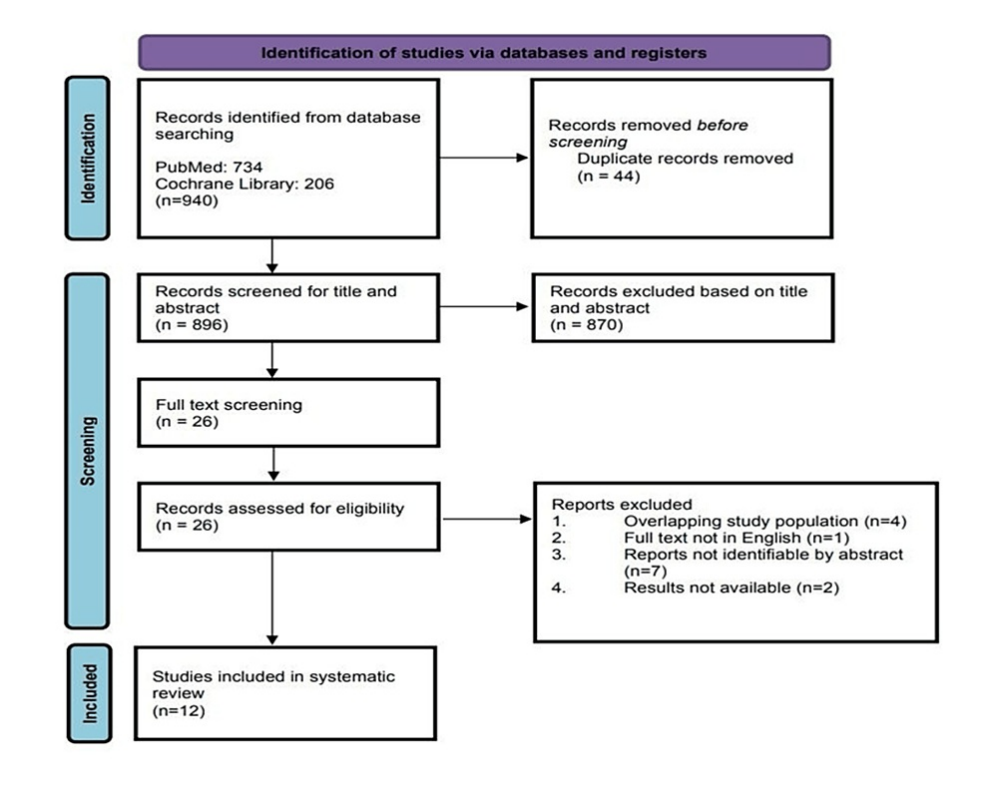
PRISMA flow diagram PRISMA: Preferred Reporting Items for Systematic Review and Meta-Analysis

Preferred Reporting Items for Systematic Review and Meta-Analysis (PRISMA) was followed to conduct this systematic review (Figure 1) [34-36].

Search Strategy and Study Selection

A systematic literature search was conducted on the PubMed and Cochrane CENTRAL databases with the various combinations of the following subject keywords and their MeSH terms: ((Holmium) OR (Holmium laser enucleation)) OR (Holmium laser enucleation of prostate)) OR (HoLEP)) AND (benign prostatic hyperplasia)) OR (BPH) OR (benign prostatic hypertrophy) by applying a time range of 2015-2022. No language restriction was applied. Results identified from the database searches were imported into EndNote 20. After duplicate results were removed, two independent reviewers carried out the title and abstract screening against the exclusion criteria. Then the full-text screening of the records that met our inclusion criteria was conducted. At any point in the screening, if two reviewers disagreed about the eligibility of an article, they discussed their assessment and sought a mutual agreement. If consensus could not be reached, a consistent third reviewer was consulted to make the final decision.

Inclusion and Exclusion Criteria

We included RCTs and original studies published from 2015 to 2022. Information needed from the articles was retrieved (population, intervention, comparison, outcome, and study). A detailed description of the PICO approach is shown in Table 1. Studies were excluded on the basis of unavailability of full text, overlapping patient populations, insufficient number of participants, incomplete data, and unavailability of final results. Furthermore, studies were excluded in which patients had neurogenic bladder, underlying prostatic malignancy, or previous surgery in the prostate. 

**Table 1:** PICO approach for defining inclusion criteria PICO: Population, intervention, comparator, outcome; BPH: benign prostatic hyperplasia

Population	Male patients with BPH, age (45-85)
Intervention	Holmium laser enucleation of the prostate (HoLEP)
Comparison	Transurethral resection of the prostate (TURP)
Outcomes	Postoperative IPSS, postvoid residual (PVR) volume, maximum flow rate (Qmax), change in sexual function

Data Extraction

Two independent reviewers extracted the data from the included studies following the PICOS (population, intervention, comparison, outcome, and study) approach. Any discrepancy in the extracted data was resolved by mutual discussion between the reviewers. Data regarding the author names, publication year and country of study, study design, participants’ baseline characteristics, outcomes, and peri- and postoperative complications were extracted from all the included studies and are mentioned in Table 2.

**Table 2:** Literature review P: Prospective, R: Retrospective, SC: Single center, MC: Multicenter, BPH: Benign prostatic hyperplasia, HoLEP: Holmium laser enucleation of prostate, TURP: Transurethral resection of prostate, TUEVP: Transurethral electrovaporization of prostate, TURIS: Transurethral resection in saline, GLPVP: Green light photoselective vaporization of the prostate, GL.PVEP: Greenlight laser Vapo‐Enucleation of the prostate, DOPC: Diagnostic outpatient cystoscopy, PVRU: Postvoidal residual urine, PSA: Prostate specific antigen, TRUS: Transrectal ultrasound, IPSS: International prostate symptom score, QoL: Quality of life score, Qmax: Maximum urinary flow rate, AUA: American Urological Association Symptom Score, IIEF-15: International Index of Erectile Function- 15, MSHQ: Ejaculatory subdomain of male sexual health, Preop: Preoperative/ly, Postop: Postoperative/postoperatively, Hb: Hemoglobin, AUR: Acute

First Author (Country, Publication year)	Study design	Total number of participants	Intervention arms and number of patients in each arm (n)	Follow-up duration	Outcomes	Peri- and post-operative complications and no. of patients (n)
Jefferson et al., (UK, 2015) [37]	P/SC	100	1. HoLEP (50) 2. TURP (50)	1. Median 66 months 2. Median 67 months	In comparison to TURP, HoLEP resulted in: Shorter hospital stay (p<0.001) More prostatic tissue removed (p<0.001) No significant difference in median (range) postoperative IPSS 4 (0–14) vs. 4 (0-23) No significant difference in median (range) postoperative QOL 1 (0–3) vs. 0 (0–5)	HoLEP vs. TURP Greater decrease in Hb levels after HoLEP (p<0.001) Median sodium decrease (− 1 vs. −1mEq/L) Repeat bladder outlet surgery (1 vs. 1) Visible hematuria requiring intervention (2 vs. 4) Urethral stricture requiring intervention (3 vs. 3) Persistent retention requiring intermittent self-catheterization (1 vs. 10)
Elshal et al., (Egypt, 2015)[38]	P/SC	245	1. HoLEP (81) 2. Monopolar TURP (112) 3. Bipolar TURP (52) 4. Sham procedure (DOPC) (35)	Six months	Baseline vs. 6 months visit Significant reduction in mean orgasm score in monopolar and bipolar groups as compared to HoLEP and Sham group Significant reduction of mean ejaculatory/MSHQ score in all groups as compared to Sham No significant changes in mean desire, erectile function, intercourse satisfaction nor overall satisfaction score in any of the groups	Not reported
Xiaofeng et al.,(China, 2016)[39]	P/SC	118	1. HoLEP (59) 2. TURP (59)	Two years	In comparison to TURP, HoLEP resulted in: Improved intraoperative and postoperative irrigation volume and time (p<0.001) Reduced catheterization time and hospital stay (p<0.001) Greater resected tissue (p< 0.001) Improved IPSS and QoL (p<0.001) Improved Qmax (p=0.012) Reduced TRUS prostate volume and obstruction (p<0.001)	HoLEP vs. TURP Lesser drop in Hb levels (p<0.001) No significant differences in change in sexual function between the groups
Song et al., (China, 2017)[40]	P/SC	120	1. TUEVP (60) 2. HoLEP (60)	3 months	In comparison to TUEVP, HoLEP resulted in: Reduced operative duration (p<0.01) Greater resected prostate weight (p<0.01) Improved intraoperative bladder irrigation duration and urinary catheter indwelling time (p<0.05) Decreased stress incontinence (p<0.05) No significant difference in IPSS and Qmax score at 3-month follow-up (p>0.05)	HoLEP vs. TUEVP Reduced intraoperative blood loss in HoLEP (p<0.01) Reduced postoperative blood loss and hospitalization duration in HoLEP (p<0.05)
Shah et al. (India, 2017) [41]	P/SC	86	1. HoLEP in patients with past TURP (43) 2. HoLEP in patients without past TURP (43)	1 year	Shorter operative time in group with prior TURP (p=0.047) Shorter postop. catherization time in group prior TURP (p=0.231) Improved AUA, Qmax and PVR in both groups at 1-, 3-, 6- and 12-months follow-up but no significant difference between the two groups	Prior TURP vs. No prior TURP Intraoperative complications: More Hb drop in no prior TURP group (p=0.484) Bleeding (1 vs. 2) Blood transfusion requirement and ureteric orifice injury (0 vs. 1) Postoperative complications: Blood transfusion and recatheterization, requirement, epididymitis, bulbar stricture (1 vs. 0) Transient incontinence (9 vs. 6) Bladder neck contracture, meatal/ submeatal stenosis (0 vs. 1) Incidental prostatic adenocarcinoma (4 vs. 6)
Jhanwar et al. (India, 2017)[42]	P/SC	144	1. TURP (72) 2.HoLEP (72)	24 months	In comparison to TURP, HoLEP resulted in: Shorter operative time (p=0.0001) Greater resected prostatic weight (p=0.03) Improved irrigation volume (p=0.0001) Improved Qmax at 12 and 24 months (p=0.004, 0.002 respectively) No significant differences in mean IPSS and PVRU between both groups	HoLEP vs. TURP More hemoglobin loss in HoLEP (p=0.08) Greater fall of serum sodium level in TURP group (p=0.0001) UTI induced fever (2 vs. 7) Postop. blood transfusion requirement (0 vs. 3) Urinary stress incontinence (2 vs. 0) No significant change in sexual function in any group
Elshal et al. (2018, Egypt)[43]	P/SC	182	1. HoLEP (60) 2. TURIS (62) 3. GL PVEP (60)	3 years	In comparison to TURIS, HoLEP resulted in: Shorter operative time (p<0.005) Greater operative efficiency (p<0.001) Greater resected tissue weight (p<0.001) Shorter hospital stay (p<0.001) Reduced catheterization time (p<0.01) No significant difference in intraoperative irrigation volume	HoLEP vs. TURIS Lesser drop in Hb levels (p<0.002) Anemia necessitating blood transfusion (4 vs. 0, p=0.03) Capsular violation (5 vs. 0, p<0.01) AUR requiring recatheterization and postoperative hematuria (0 vs. 1 and 4 vs. 3 respectively) Recurrent obstructive LUTs (2 vs. 11, p=0.01) LUTs with residual prostate adenoma (0 vs. 6, p<0.04) Bladder neck contracture (1 vs.0) Persistent urge urine incontinence (6 vs. 6)
Sinha et al. (India, 2019)[44]	P/MC	144	1. HoLEP (72) 2. TURP (72)	24 months	In comparison to TURP, HoLEP resulted in: Longer operative time (p=0.0001) Greater mean resected tissue (p= 0.03) Shorter hospital stay	In comparison to TURP, HoLEP resulted in: Higher postop. dysuria occurrence Less blood loss Lower rate of transfusion requirement No significant change in sexual function in postop. follow-up
Bai et al. (China, 2019) [45]	P/SC	65	1. HoLEP (33) 2.TURP (32)	3 days	In comparison to TURP, HoLEP resulted in: Longer operative time (p<0.0001) Greater resected prostatic weight (p=0.001) Reduced bed rest (p<0.04) Reduced catheterization time (p<0.031) Shorter hospital stay (p<0.0001)	1 day after procedure, PF1+2 and TAT, t-PA, and PAI-1 elevated significantly from baseline (p<0.05) Lower PF1+2 and TAT levels in HoLEP group (p<0.05) No significant differences in t-PA and PAI-1 levels between two groups
Prudhomme et al. (France, 2020) [46]	R/MC	60	1. TURP (34) 2. HoLEP (17) 3. GL PVP (9)	12 months	In comparison to TURP, HoLEP resulted in: Shorter operative time (p=0.04) Resected prostatic weight (p=0.3) Irrigation volume (p=0.0001) Shorter hospital stay (p<0.0001)	HoLEP vs. TURP Greater drop in Hb levels in HoLEP group (p=0.1) Early postop complications: Anemia requiring blood transfusion (1 vs. 0) UTI (0 vs. 1) AUR requiring bladder catheterization (4 vs. 7) One-year postop complications: Anemia requiring blood transfusion (1 vs. 0) UTI (5 vs. 0, p=0.001) AUR requiring bladder catheterization (5 vs. 1, p=0.01)
Hassan et al. (Egypt, 2020)[47]	P/SC	60	1. HoLEP (30) 2. Bipolar TURP (30)	12 months	In comparison to TURP, HoLEP resulted in improved PVRU, and Qmax at 12 months postoperatively, shorter hospital stay and catheterization time (p=0.7)	Lesser mean Hb loss in HoLEP No significant difference in postoperative complications between both groups
El-Hawy et al. (Egypt, 2021)[48]	P/SC	114	1. Bipolar HoLEP (59) 2. TURP (55)	24 months	In comparison to bipolar TURP, HoLEP resulted in: Reduced catheterization time (p=0.001) Shorter hospital stay (p=0.016) Longer operative time (p=0.005) Greater resected tissue weight (p=0.007) Improved mean IPSS score (p=0.004, 0.002, 0.002 at 3,6 and 12 months) Improved mean Qmax (p=0.02 at 12 months) No significant differences in irrigation volume, improved IPSS at 1-month, Qmax at 1,3,6, 24- months, PVRU at 6 and 12-months between both groups	HoLEP vs. TURP Less drop in Hb levels in HoLEP group (p=0.11) Drop in serum sodium levels (p=0.14) Intraoperative bleeding and blood transfusion (0 vs. 1) Capsular injury (6 vs. 7) Bladder injury (3 vs. 0) UTI (4 vs. 5) Urge incontinence (10 vs. 8)

Risk of Bias Assessment

**Figure 2:**
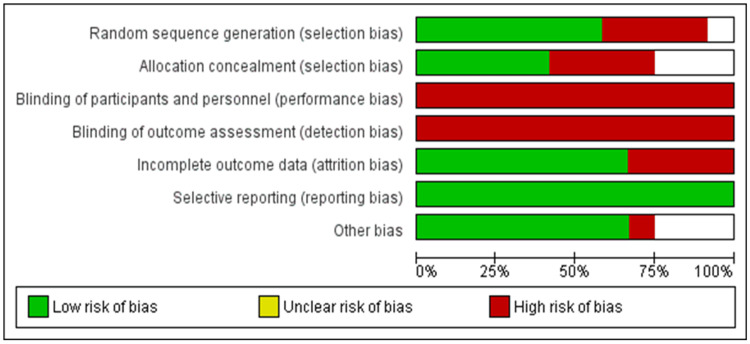
Risk of bias graphs of the included studies

**Figure 3:**
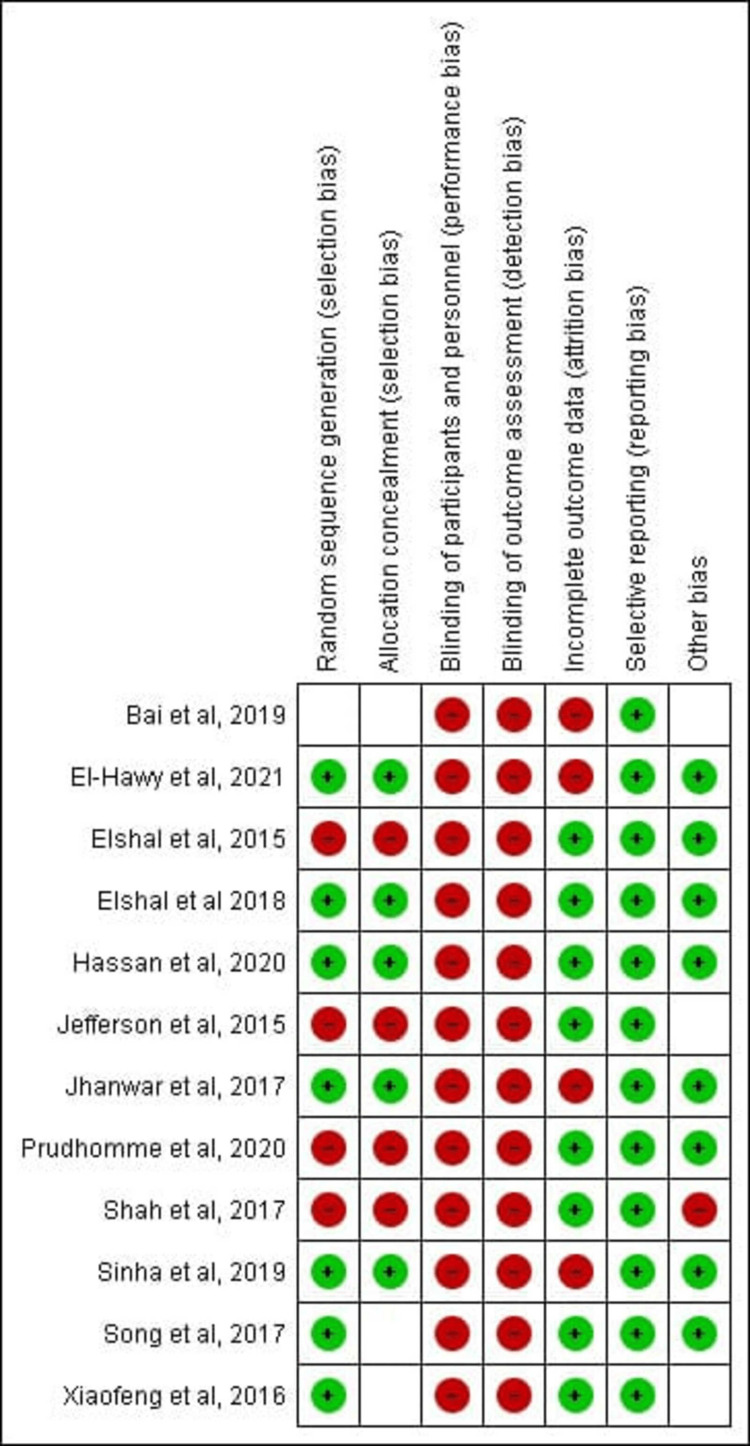
Risk of bias summary of the included studies References: [37-48]

The quality assessment of selected studies was done using the Cochrane collaboration risk of bias assessment tool [35,36], as shown in Figures 2, 3. The selection bias, reporting bias, performance bias, detection bias, attrition bias, and other biases were assessed for each study. We rated the risk of bias for each study as low, high, or unclear. In a large number of the included trials, the risk of performance bias was high as blinding the surgeons, investigators, and patients was not feasible. Most of the studies showed a low risk of selection, attrition, and reporting bias [35].

Results

An initial systematic literature search was carried out on two electronic databases i.e., PubMed and Cochrane Central using various keywords and their MeSH terms. We found 734 studies on PubMed and 206 studies in Cochrane Library. Duplicate records were removed and the articles were screened for title and abstracts. It yielded 26 studies for full-text screening. Finally, articles were assessed for eligibility and only 12 studies were included in the systematic review. This can be seen in the PRISMA flowchart.

All 12 studies had male participants in the age range of 45-85 years and has BPH. A comparison was made between HoLEP and TURP for prostate treatment in all the studies. Primary outcomes were postoperative international prostate symptom score (IPSS), postvoid residual (PVR) volume, maximum flow rate (Qmax), and change in sexual function following treatment.

Operative Time and Hospital Stay

While comparing TURP with HoLEP, HoLEP resulted in a short hospital stay as reported by Jefferson et al., Elshal et al., Sinha et al., Bai et al., Prudhomme et al., and El-Hawy et al. HoLEP also resulted in reduced operative time as compared to TURP, as reported by Song et al., Shah et al., Jhanwar et al., and Elshal et al. In studies conducted by Sinha et al. and Bai et al., HoLEP resulted in longer operative time as compared to TURP [36-48].

Postoperative International Prostate Symptom Score (IPSS)

In comparison to TURP, HoLEP resulted in an improved postoperative IPSS as reported by studies conducted by Xiaofeng et al. [39], Hassan et al. [47], and El-Hawy et al. [48]. In contrast to that, no significant difference was observed in the mean postoperative IPSS when comparing TURP versus HoLEP in studies conducted by Jefferson et al. [37], Song et al. [40], and Jhanwar et al. [42].

Maximum Flow Rate

In comparison to TURP, HoLEP resulted in an improved maximum flow rate (Qmax) in the postoperative period as reported by Xiaofeng et al. [39], Shah et al. [41], Jhanwar et al. [42], Hassan et al. [47], and El-Hawy et al. [48]. However, a study conducted by Song et al. concluded no significant difference in the Qmax score following HoLEP in BPH patients [40].

PVR Urine Volume

The PVR volume was improved in patients following HoLEP as reported by Shah et al. [41], Hassan et al. [47], and El-Hawy et al. [48]. However, no significant change was observed in the PVR volume in patients following TURP or HoLEP as reported by Jhanwar et al. [42].

Changes in Sexual Functions

A significant reduction in the mean orgasm score was observed in patients following monopolar or bipolar TURP as reported by Elshal et al. [38]. The mean ejaculatory score was also reduced in patients following HoLEP and TURP but no significant change in erectile function, intercourse satisfaction, and overall satisfaction score was observed as reported by Elshal et al. [38].

Discussion

When it comes to HoLEP, it results in a shorter duration of hospital stay, as mentioned in studies by Jefferson et al. [37], Elshal et al. [43], Sinha et al. [44], Bai et al. [45], Prudhomme et al. [46], and El-Hawy et al. [48]. This is supported by a recent systematic review on the same topic [31]. HoLEP is also associated with a shorter operative time, as reported by Song et al. [40], Shah et al. [41], Jhanwar et al. [42], and Elshal et al. [43]. However, there are findings of a longer operative time for HoLEP compared to Transurethral Resection of the Prostate (TURP), as reported by Sinha et al. [44] and Bai et al. [45], which aligns with the results of a recent systematic review on the same topic [31]. This suggests that TURP may be superior to HoLEP. These results are supported by a study [49] where a shorter operative time was favored for TURP. According to a study, this longer operative time is attributed to the steep learning curve associated with HoLEP [50], as well as variations in the surgeon's skill set. However, this steep learning curve can be overcome with a structured training program and proper supervision during the learning process [51].

HoLEP also resulted in improved postoperative International Prostate Symptom Score (IPSS), as reported by studies conducted by Xiaofeng et al. [39], Hassan et al. [47], and El-Hawy et al. These results are supported by Zhong et al. [31], where the 12-month follow-up showed that HoLEP yielded better IPSS scores compared to TURP. However, the same study [31] also stated that the 1-month, 6-month, and 24-month follow-ups showed no significant difference in IPSS scores between HoLEP and TURP, as reported by Jefferson et al. [37], Song et al. [40], and Jhanwer et al. [42].

The PVR volume improved in patients following HoLEP, as reported by Shah et al. [41] and Hassan et al. [47], consistent with findings in another recent systematic review [31,52]. According to this recent review [52], post-residual volume at the 12-month follow-up was superior in HoLEP patients compared to TURP.

Moreover, HoLEP also resulted in an improved maximum flow rate (Qmax) in the postoperative period, as reported by Xiaofeng et al. [39], Shah et al. [41], Jhanwar et al. [42], Hassan et al. [47], and El-Hawy et al. [48], which is supported by a study [50] where peak urine flow increased after HoLEP. A better maximum flow rate was associated with HoLEP at the 18- and 24-month follow-ups when compared with TURP [31]. However, this study's findings are contradicted, as follow-up at 6 and 12 months showed no significant difference in Qmax scores following HoLEP, which is also supported by Song et al. [40] in our review.

Previous reviews and meta-analyses [31] were limited in their data regarding the effects of HoLEP and TURP on sexual dysfunction. Our review concluded that a significant reduction in mean orgasm score was observed in patients following monopolar or bipolar TURP, as reported by Elshal et al. [38]. However, there was no significant change in erectile function, intercourse satisfaction, and overall satisfaction scores, as reported by Elshal et al. [38]. A prospective study also concluded that there was no significant sexual dysfunction in the post-surgical period for both HoLEP and TURP patients [53].

Higher tissue retrieval is also seen with HoLEP, resulting in a higher prevalence of detection of incidental prostate cancers during HoLEP [54]. This further establishes HoLEP's superiority to TURP because the detection of incidental cancers is significant, even in patients with a prostate-specific antigen (PSA) level of less than 10 ng/ml, and these cancers are often detected after HoLEP [55].

Moreover, HoLEP is considered superior when patients experience bleeding complications. It has been proven to offer better hemostatic control, as supported by our review. It is also associated with decreased drops in hemoglobin levels, reduced blood loss, and fewer side effects during the perioperative period [56]. In contrast, TURP has been associated with more activation of thrombin generation, a higher thrombotic tendency, and is linked to a hypercoagulable state [45].

Historically, TURP had been the gold standard, as indicated in the systematic review in 2019 [31,49]. However, TURP is still associated with a high risk of morbidity and mortality. This is why it has been replaced by the size-independent gold standard for the treatment of benign prostatic hyperplasia (BPH), which is HoLEP [57], due to its superior efficacy in the postoperative period [8]. Although HoLEP was historically considered suitable for patients with prostate volumes less than 100 mL/100 g, it has evolved to become a size-independent gold standard for BPH treatment [52].

Limitation

One notable limitation in the existing literature is the dearth of comprehensive data regarding the effects of HoLEP and TURP on sexual dysfunction, not all studies were randomized, and these studies have impact performance and detection bias. Given the importance of this aspect in the overall patient experience, further research in this area is warranted to provide a more comprehensive assessment of these treatment modalities. 

## Conclusions

HoLEP demonstrates promising advantages over TURP in various aspects of prostate treatment. Notably, it is associated with a shorter hospital stay for a substantial number of patients and, in some cases, a reduced operative time. While there were isolated instances of longer operative times with HoLEP, the overall trend suggests efficiency and effectiveness.

In terms of postoperative outcomes, HoLEP appears to offer improvements in several crucial parameters. The postoperative IPSS showed notable enhancements in a subset of patients undergoing HoLEP, which could potentially translate to a better quality of life post-treatment. Similarly, an improved Qmax was observed in a significant number of HoLEP patients, indicating enhanced urinary flow. Additionally, HoLEP contributed to the reduction of the PVR volume in certain cases, further highlighting its positive impact on urinary function.
